# Neurofilament Light Chain in Cerebrospinal Fluid and Blood in Multiple System Atrophy: A Systematic Review and Meta-Analysis

**DOI:** 10.3390/brainsci15030241

**Published:** 2025-02-25

**Authors:** Silvia Demiri, Dimitra Veltsista, Vasileios Siokas, Kanellos C. Spiliopoulos, Antonia Tsika, Polyxeni Stamati, Elisabeth Chroni, Efthimios Dardiotis, Ioannis Liampas

**Affiliations:** 1 Department of Neurology, University Hospital of Patras, School of Medicine, University of Patras, 26504 Patras, Greece; sylviantemiri@gmail.com (S.D.); dveltsista@yahoo.gr (D.V.); k.spiliopoulos@ac.upatras.gr (K.C.S.); echroni@upatras.gr (E.C.); 2 Department of Neurology, University Hospital of Larissa, School of Medicine, University of Thessaly, 41100 Larissa, Greece; vsiokas@med.uth.gr (V.S.); antonellatsi@hotmail.com (A.T.); tzeni_0@yahoo.gr (P.S.); edar@med.uth.gr (E.D.)

**Keywords:** Parkinson’s disease, Lewy body dementia, progressive supranuclear palsy, corticobasal syndrome, corticobasal degeneration

## Abstract

**Background/Objectives**: Multiple system atrophy (MSA) presents a challenging diagnosis due to its clinical overlap with other neurodegenerative disorders, especially other α-synucleinopathies. The main purpose of this systematic review and meta-analysis was to assess neurofilament light chain (NfL) differences in the CSF and blood of patients with MSA versus the healthy control group (HC), patients with Parkinson’s disease (PD) and patients with Lewy body dementia (LBD). Secondarily, the diagnostic metrics of CSF and circulating NfL in MSA versus HC, PD, LBD, progressive supranuclear palsy (PSP) and corticobasal degeneration (CBD) were discussed. **Methods**: MEDLINE and EMBASE were thoroughly searched for relevant case-control studies. Standardized mean differences (SMDs) were calculated separately for CSF and blood NfL per comparison. Statistical heterogeneity was assessed based on the Q and I^2 statistics. **Results**: Twenty-five relevant studies were retrieved. Quantitative syntheses revealed elevated CSF and circulating NfL levels in individuals with MSA versus HC [SMD = 1.80 (95%CI = 1.66, 1.94) and SMD = 2.00 (95%CI = 1.36, 2.63), respectively] versus PD [SMD = 1.65 (95%CI = 1.26, 2.03) and SMD = 1.63 (95%CI = 0.84, 2.43), respectively] as well as versus LBD [SMD = 1.17, (95%CI = 0.71, 1.63) and SMD = 0.65 (95%CI = 0.30, 1.00), respectively]. Diagnostic accuracy was outstanding for CSF and blood NfL in MSA versus HC and PD, and it was moderate in MSA versus LBD. On the other hand, it was suboptimal in MSA vs. PSP and CBD. **Conclusions**: Both CSF and circulating NfL levels are elevated in MSA compared to HC, PD and LBD. To achieve optimal diagnostic properties, further work is required in the standardization of processes and the establishment of reference NfL intervals and/or thresholds.

## 1. Introduction

Multiple system atrophy (MSA) is a sporadic, adult-onset, progressive neurodegenerative disorder, which is characterized by clinical features of autonomic dysfunction, parkinsonism, and/or cerebellar ataxia [1]. The disease typically manifests in the 6th decade of life with a worldwide yearly incidence of fewer than 5 cases per 100,000 people and an average survival time of 6–10 years from symptom onset [2,3]. MSA is classified into two main phenotypes depending on the predominance of either parkinsonian symptoms (MSA-P) or cerebellar ataxia (MSA-C). The clinical MSA subtypes correlate with neuropathological findings of degeneration in the striatonigral and olivopontocerebellar regions, respectively. Although the underlying pathophysiological mechanisms have yet to be deciphered, studies indicate that glial cytoplasmic inclusions (GCIs) in oligodendrocytes—mainly composed of α-synuclein aggregates—are pivotal in the cascade toward neuronal death and reactive gliosis [4].

The diagnosis of MSA often poses a significant challenge for clinicians due to its complex nature and overlap with other movement disorders, particularly Parkinson’s disease [5]. Therefore, objective and accurate diagnostic biomarkers are needed to prevent diagnostic misclassification and ensure the correct identification of patients with MSA. Researchers have already explored the diagnostic utility of multiple imaging and fluid biomarkers [4]. With respect to fluid biomarkers, neurofilament light chain (NfL) has probably received the greatest focus, with α-synuclein, tau, phosphorylated tau (p-tau), and glial fibrillary acidic protein (GFAP) following [6].

NfL is an intermediate filament and a key component of the neuronal cytoskeleton. Its concentration sharply increases during neuronal axonal damage and can be quantified in both CSF and blood samples [7,8]. NfL has been suggested as a potential biomarker in various neurodegenerative disorders including multiple sclerosis (MS), Alzheimer’s disease (AD), frontotemporal dementia (FTD), amyotrophic lateral sclerosis (ALS) and Parkinsonian disorders [9,10,11]. In this systematic review and meta-analysis, we emphasized the potential diagnostic utility of NfL in MSA. Cerebrospinal fluid (CSF) and circulating NfL were separately investigated. Our primary focus was the use of NfL in the discrimination of individuals with MSA from the healthy control group (HC), patients with Parkinson’s disease (PD) and Lewy body dementia (LBD: PD dementia plus dementia with Lewy bodies—DLB). Secondarily, we assessed its diagnostic properties in MSA vs. progressive supranuclear palsy (PSP) and corticobasal degeneration/syndrome (CBD/S).

### 1.1. Historical Overview of MSA

The term ‘multiple system atrophy’ was first introduced by J.G. Graham and D.R. Oppenheimer in 1969 in their published study “Orthostatic hypotension and nicotine sensitivity in a case of multiple system atrophy”. However, several syndromes that fall within the spectrum of the disease had already been described in the literature. Below, we provide a brief overview of the historical milestones of the disease [4].

The first description of MSA dates back to 1900 in the Salpêtrière Hospital in Paris, where J. Dejerine and A. Thomas reported two cases with ataxia, dysarthria, extrapyramidal symptoms, and autonomic dysfunction using the term olivopontocerebellar atrophy (OPCA) [12]. In 1925, Bradbury S. and Eggleston C. from Cornell University in New York presented three cases with autonomic dysfunction suggesting idiopathic origin and soon several published reports associating autonomic failure with extrapyramidal manifestations [13]. Later, in 1960, G.M. Shy from the NIH and G.A. Drager from Baylor College of Medicine in Houston described an eponymous syndrome associated with marked autonomic failure, parkinsonian, cerebellar, and pyramidal signs. Shy–Drager syndrome was initially viewed as a separate entity; now it is placed under the umbrella of MSA [12]. In 1960 and 1961, Van der Eecken and R.D. Adams provided clinical and neuropathological findings through their research regarding striatonigral, cerebellar, pontine and olivary degeneration, which is now identified as MSA atrophy loci [13].

As previously stated, Graham and Oppenheimer in Oxford coined the term MSA for the aforementioned conditions in 1969. Two decades later, Papp, Kahn and Lantos supported the unified classification by providing distinct histopathological findings from MSA patients’ brains—GCIs or Papp–Lantos inclusions—proposing that myelin degeneration precedes atrophy [14]. In 1997, M.G. Spillantini identified α-synuclein as the constituent of Lewy bodies and GCIs [4]. Histopathological findings of GCIs are now established as criteria for the definite diagnosis of the disorder [1].

### 1.2. Neuropathology of MSA

The exact etiology of MSA remains unknown. The neuropathological hallmark of MSA is the presence of GCIs, which are primarily composed of insoluble α-synuclein aggregates. Since α-synuclein is not typically produced in healthy oligodendrocytes and similar inclusions can also be identified in glial nuclei, neuronal cells and astroglia cells, the origin of oligodendrocytic α-synuclein remains unclear [15]. Published evidence supports a prion-like propagation of α-synuclein [16]. Metabolic (mitochondrial dysfunction) and oxidative stress are believed to play a crucial role in the pathogenesis, as mutations in the COQ2 gene encoding coenzyme 10 have been implicated [17]. Neuroinflammation, myelin degeneration, astrogliosis and neuronal loss constitute the sequalae of misfolded α-synuclein accumulation and can be histologically identified postmortem [17]. Macroscopically, there is atrophy and discoloration in the striatonigral system (dorsolateral putamen, caudate, substantia nigra) and olivopontocerebellar regions (cerebellar folia, pontine basis and middle cerebellar peduncle) [4,18].

### 1.3. Clinical Manifestations in MSA

The presenting symptoms can vary among MSA patients. Autonomic dysfunction is reported first in most cases with erectile dysfunction being the earliest symptom and hypotension exhibiting the strongest associations with incident MSA [19]. Patients may also report urinary dysfunction manifesting as overactive bladder and incomplete bladder emptying [20]. Although autonomic symptoms are also common in PD, in MSA, they tend to present earlier and be much more debilitating [12]. The early progression of autonomic failure (within 2.5 years of diagnosis) has been documented as an independent risk factor for poorer prognosis, shorter time to becoming wheelchair-bound and shorter survival [21].

Regarding the motor manifestations, there are two main motor phenotypes: MSA-P and MSA-C. The former type usually presents with signs of rigidity, bradykinesia, tremor or postural instability. The latter is typically related to gait ataxia, which is often accompanied by limb ataxia, dysarthria and oculomotor dysfunction [22]. Of note, pyramidal symptoms are documented in approximately half of the patients and are associated with degeneration of the pyramidal tracts [18]. Clinical presentations of MSA can include overlaps between MSA-P and MSA-C. Unfortunately, misdiagnosis due to motor manifestations is common. Koga and colleagues reported that clinically diagnosed cases of MSA are often misclassified as LBD, PSP and PD [23]. Therefore, identifying objective diagnostic biomarkers is of utmost importance to prevent misdiagnosis.

Apart from the prominent motor and autonomic dysfunction, MSA additionally presents with REM sleep behavior disorder (RBD) and sleep apnea. Notably, in the vast majority, sleep disorders predate motor symptoms [4]. Cognitive deficits have also been documented in several patients; however, early dementia onset should raise suspicion of alternative diagnoses [1].

### 1.4. Neurofilament Light Chain

Neurofilaments (NFs) are components of the axonal cytoskeleton, expressed exclusively in neurons, contributing to their structure and regulating intracellular and axonal transport. Four different subunits of NF are encoded in the central nervous system: heavy (NFH), medium (NFM), light (NFL) NF and α-internexin. NFs have been reported to increase with aging and neurodegeneration; the best studied and most promising among these subunits is NfL. NfL expression is particularly high in large, myelinated axons and under normal conditions; low levels of NfL are constantly released due age-related neuronal turnover [7,11]. In neuroaxonal damage, NfL increases markedly in the interstitial fluid, subsequently passing into the CSF and entering the bloodstream [10,24]. Even though NfL levels are higher in CSF than in blood, both measurements may hold a biomarker value [8]. Elevated concentrations of NfL have been reported in several neurodegenerative disorders including MS, AD, FTD, ALS and Parkinsonian disorders [9,25]. NfL is also being investigated as a potential biomarker of psychiatric conditions such as bipolar disorder (BD), major depressive disorder (MDD) and psychotic disorders [26].

## 2. Materials and Methods

This review adheres to the Preferred Reporting Items for Systematic Reviews and Meta-Analyses (PRISMA) guidelines (Supplementary Materials). All study procedures were performed by two authors, independently (S.D. and D.V.). Discrepancies were resolved by a third author (I.L.).

### 2.1. Search Methods

A systematic literature search of the MEDLINE (through PubMed) and EMBASE (through Elsevier) databases was performed to retrieve every relevant study published from inception to December 2024 (final literature search). The following terms were applied (throughout the text): [“Multiple System Atrophy” OR “MSA”] AND [“CSF biomarkers” OR “blood biomarkers” OR “neurofilament light chain” OR “NfL”]. Finally, every retrieved paper, as well as every relevant systematic review and meta-analysis, was manually scrutinized for relevant references.

### 2.2. Eligibility Criteria

Studies were included if they met the following criteria:

- Published before December 2024;
- Designed as case-control studies (irrespective of the study design being retrospective or prospective);
- Including at least 2 groups: one group of patients with MSA and one group of HC or patients with PD, LBD, PSP, CDB;
- Measured NfL in CSF and/or blood samples and/or investigating NfL’s diagnostic properties.

Studies were excluded according to the following criteria:

- Studies assessing alternative biomarkers, not involving NfL;
- Studies assessing NfL’s prognostic—and not diagnostic—properties;
- Studies assessing alternative disease groups, not involving MSA or any one of the well-defined comparators;
- Other study designs (review, meta-analysis, etc.);
- Studies not involving human subjects;
- Studies not published in English;
- Irrelevant papers;
- Study protocols;
- Retracted publications.

All titles and abstracts were manually screened for eligibility. Full texts of the studies that qualified from the initial screening were reviewed to establish if an article fulfilled the inclusion criteria. Eligible studies were involved in the qualitative analysis and, if appropriate, in the quantitative synthesis of the results.

### 2.3. Data Extraction—Outcome Measures

Data were extracted based on standardized data extraction forms: first author, country of origin, publication year, study design and settings, method of NfL measurement, set of diagnostic criteria, number of participants, age and sex distribution, disease duration and clinical rating scales (e.g., Hoehn and Yahr scale, Unified Parkinson’s Disease Rating Scale [UPDRS-III]). Then, the mean values and standard deviations (SD) of NfL concentrations in CSF and/or blood were obtained. When only median, minimum and maximum values were provided, mean and SD were calculated based on Hozo and colleagues [27]. If median, Q1 and Q3 values were available, mean and SD were estimated based on Wan and colleagues [28]. Where median and interquartile range values were reported (without Q1 and Q3 values), mean and SD were calculated according to Greco and colleagues [29].

Our primary outcome was the standardized mean difference (SMD) in NfL levels between MSA and (1) HC, (2) PD and (3) LBD, to assess the potential diagnostic applicability of NfL. Our secondary focus was NfL differences in MSA vs. (1) PSP and (2) CBD/S. These entities are less prevalent and constitute a rarer and less important diagnostic challenge (α-synucleinopathies share autonomic along with parkinsonian features that pose the main diagnostic challenge). In addition, the promising diagnostic properties of α-synuclein real-time quaking-induced conversion in the distinction of α-synucleinopathies from PSP and CBD will probably make the use of alternative biomarkers redundant in the differentiation of these conditions. Therefore, we only performed a qualitative synthesis. Moreover, as a secondary measure, we discuss the diagnostic metrics of NfL measurements in CSF and blood: area under the curve (AUC–correct classification), sensitivity and specificity.

### 2.4. Statistical Analysis

RevMan 8.13.0 statistical software was utilized for all statistical analyses (https://revman.cochrane.org/info, last accessed on 1 January 2025). The level of statistical significance was conventionally set to α = 0.05 (*p* < 0.05). SMDs (effect sizes in relation to study variability) and their precision (95% confidence intervals [95%CIs]) were estimated for circulating and/or CSF NfL using as weights the inverse variance of individual effects. Heterogeneity among trials was statistically evaluated according to the Q and I^2 statistic (more appropriate for a small number of studies). Homogeneity was rejected if PQ < 0.1 or $I^{2}$ > 50%. A fixed-effects (FE) model was used if homogeneity was confirmed; otherwise, a random-effects (RE) model was applied. SMDs and 95%CIs were graphically depicted with forest plots. Publication bias was assessed with funnel plots when 10 or more studies were synthesized.

## 3. Results

### 3.1. Literature Search

**Figure 1:**
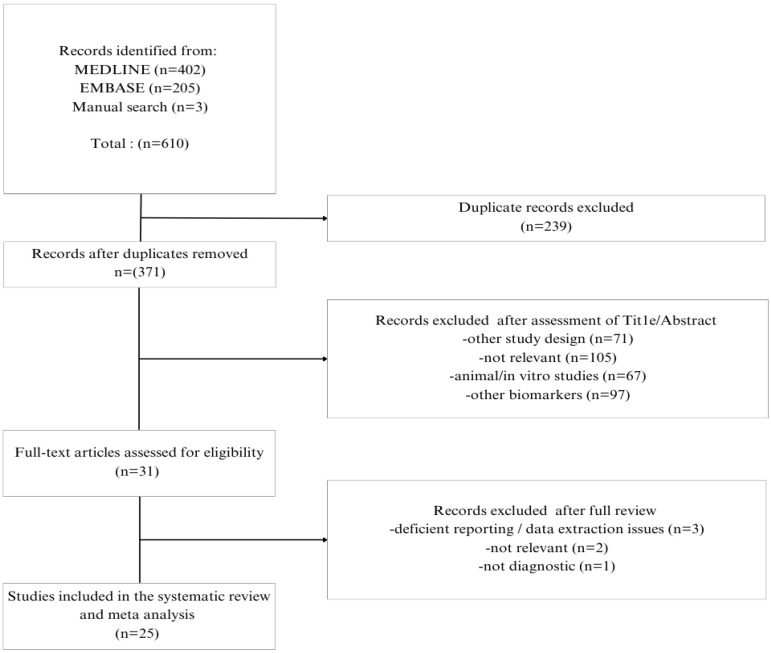
Study selection flow chart.

The literature search yielded 610 studies, 402 from MEDLINE (through Pubmed), 205 from EMBASE (through Elsevier), and 3 from the manual search. After the initial assessment of titles and abstracts, 31 full texts were evaluated for eligibility. Finally, 25 studies were included in this systematic review and 24 of them were included in the quantitative syntheses (Figure 1).

### 3.2. Study Characteristics

A total of 25 studies published between 1998 and 2024 were included in this systematic review and meta-analysis. The majority of the eligible studies originated from Sweden (*N* = 7) [30,31,32,33,34,35] and China (*N* = 5) [36,37,38,39,40]. The remaining studies were conducted in the Netherlands [41,42,43], Germany [44,45,46], England [47], Italy [48], Denmark [49], Japan [50], Taiwan [51], Finland [52] and the USA [53].

There were 23 studies involving HC and an even number of articles on individuals with PD. Fewer studies included participants with LBD (*N* = 6), PSP (*N* = 14) and CBD/S (*N* = 7). The vast majority of MSA participants were in their 6th or 7th decade of life, and biomarker investigations were conducted after an average disease duration of 2 to 7 years. Among the retrieved articles, CSF NfL was examined by 14 studies [30,31,32,33,34,41,43,47,49,50,52,53,54], circulating NfL by 6 studies [36,37,38,40,51] and both CSF and blood NfL by 5 studies [42,44,45,46,48]. Detailed information on the settings and diagnostic processes, as well as participant characteristics (demographics, disease duration and severity), are in Table 1 (based on study reporting). Study results on the comparisons between MSA and HC, PD and/or LBD (footnotes) are provided in detail in Table 2.

#### 3.2.1. NfL in MSA vs. HC

**Figure 2:**
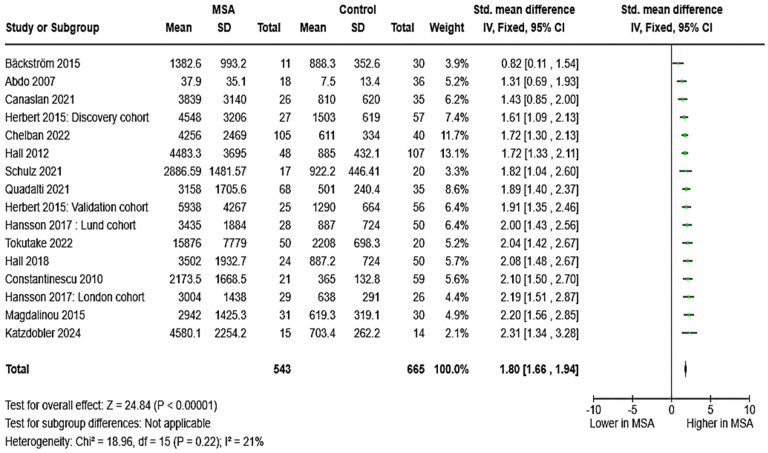
CSF levels of NfL in patients with multiple system atrophy (MSA) vs. controls [31,32,34,35,41,43,44,45,46,47,48,50,52,54].

The levels of CSF NfL were compared in 14 studies (two of which featured two cohorts) involving a total of 543 patients with MSA and 665 HCs. CSF NfL levels were consistently elevated in participants with MSA across all 14 studies. The estimated pooled effect was SMD = 1.80, [95%CI = (1.66, 1.94)] (Figure 2). Data on diagnostic metrics of CSF NfL (*N* = 3) suggested an excellent, almost perfect diagnostic accuracy in distinguishing MSA from HC: AUC values ranged from 0.925 to 1.000. Of note, substantial differences were reported by different authors regarding optimal cut-points (ranging from 1024 to 3827 pg/mL).

**Figure 3:**
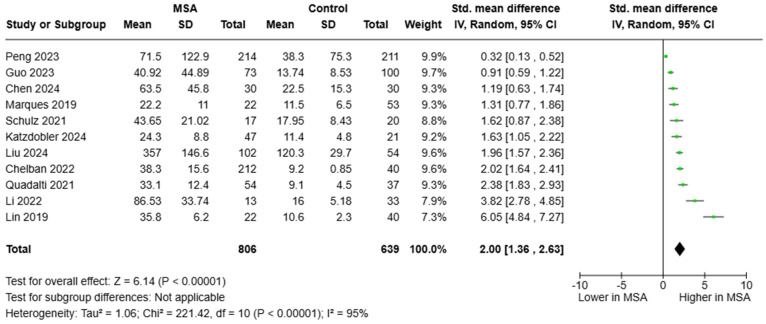
Blood levels of NfL in patients with multiple system atrophy (MSA) vs. controls [36,37,38,39,40,42,44,45,46,48,51].

Regarding circulating NfL, we analyzed 11 studies that included a total of 806 individuals with MSA and 639 HCs. Again, all studies provided consistent findings, indicating elevated blood NfL in individuals MSA compared to HCs. The estimated pooled effect was SMD = 2.00, 95%CI = (1.36, 2.63)] (Figure 3). Six studies reported data on the diagnostic metrics of blood NfL in separating patients with MSA from HCs. Notably, circulating NfL appeared very promising, exhibiting an excellent, almost perfect diagnostic accuracy and yielding diagnostic metrics similar to those of CSF NfL (AUC ranged between 0.916 and 1.000). Although only a fraction of the studies reported optimum cut-offs, plasma NfL levels between 21.5 and 22.7 pg/mL distinguished between MSA and HCs.

#### 3.2.2. NfL in MSA vs. PD

**Figure 4:**
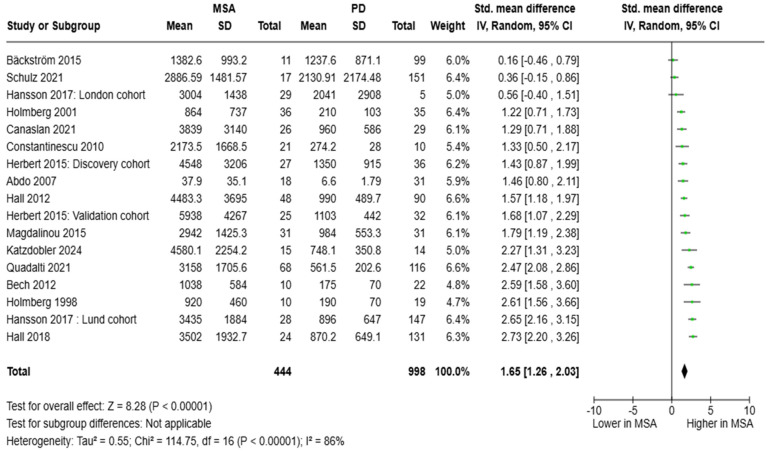
CSF levels of NfL in patients with multiple system atrophy (MSA) vs. Parkinson’s disease (PD) [30,31,32,33,34,35,41,43,44,45,47,48,49,52,54].

Regarding CSF NfL, 15 studies (two of which featured two cohorts) including a total of 444 patients with MSA, and 998 with PD were pooled. CSF NfL concentrations were found elevated in MSA compared to PD: SMD = 1.65 [95%CI = (1.26, 2.03)] (Figure 4). With respect to diagnostic metrics, one study reported an AUC of 0.712 (moderate diagnostic accuracy); however, three studies found an AUC ranging from 0.920 to 0.991, which was consistent with an almost perfect diagnostic accuracy. CSF NfL values ranging from 1196 to 1835 pg/mL distinguished between the two entities. Of note, one study grouping patients with PD and LBD together vs. MSA (*N* = 1, AUC = 0.970) reported optimal diagnostic metrics for CSF NfL.

**Figure 5:**
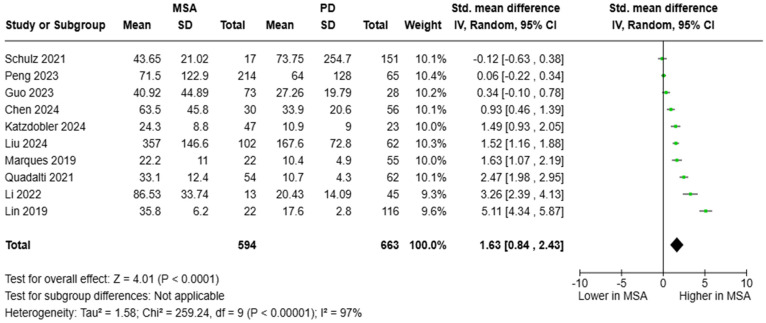
Blood levels of NfL in patients with multiple system atrophy (MSA) vs. Parkinson’s disease (PD) [36,37,38,39,40,42,44,45,48,51].

Data from 10 studies were pooled to estimate the SMD of blood NfL between patients with MSA and PD. A pooled cohort of 594 individuals with MSA and 663 with PD was formed. Circulating NfL levels were significantly elevated in MSA [SMD = 1.63, 95%CI = (0.84, 2.43)] (Figure 5). Diagnostic metrics were documented in 10 articles. AUC values ranged between 0.617 and 0.983; however, the vast majority of studies (*N* = 8) reported an AUC value > 0.800, which is indicative of an outstanding diagnostic accuracy. Plasma NfL levels between 14.1 and 24.4 pg/mL were reported to distinguish between MSA and PD.

#### 3.2.3. NfL in MSA vs. LBD

**Figure 6:**
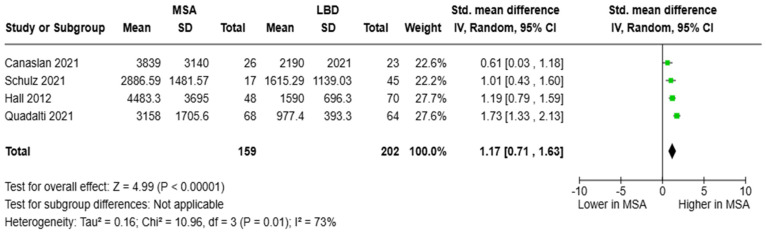
CSF levels of NfL in patients with multiple system atrophy (MSA) vs. Lewy body dementia (LBD) [32,45,48,52].

The SMD of CSF NfL levels between patients with MSA (*N* = 159) and LBD (*N* = 202) was pooled from four studies. The analysis revealed that NfL concentrations in those with MSA were higher than in those with LBD: [SMD = 1.17, 95%CI = (0.71, 1.63)] (Figure 6). Only Schulz and colleagues estimated the diagnostic metrics of CSF NfL in the distinction between MSA and LBD, reporting an AUC of 0.771 (cut-off value was not reported) [45]. Of note, Singer and colleagues compared individuals with MSA vs. LBD/PD and reported an AUC of 0.97 with an optimum threshold of 1400 pg/mL for CSF NfL [53].

**Figure 7:**
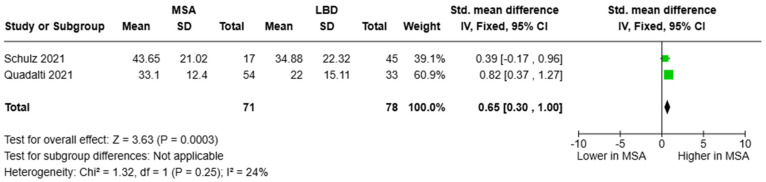
Blood levels of NfL in patients with multiple system atrophy (MSA) vs. Lewy body dementia patients (LBD) [45,48].

As for circulating NfL, the studies of Schulz and colleagues [45] as well as Quadalti and colleagues [48] were pooled, resulting in a cohort of 71 participants with MSA patients and 78 with LBD. Blood NfL concentrations were higher in MSA than in LBD [SMD = 0.65, 95%CI = (0.30, 1.00)] (Figure 7). Only Schulz and colleagues provided relevant diagnostic metrics with an AUC of 0.661 (cut-off value was not reported) [45].

#### 3.2.4. NfL in MSA vs. PSP/CBD/S

Overall, CSF and circulating NfL differences in MSA versus PSP or CBD/S do not adhere to any specific pattern with the vast majority of articles not revealing any significant differences [32,39,42,45,47].

Regarding the diagnostic performance of CSF NfL in relation to MSA and PSP, we retrieved four relevant studies. Magdalinou and colleagues [47] as well as Schulz and colleagues [45] reported relatively poor diagnostic metrics (AUCs of 0.66 and 0.577, respectively) in the differentiation between MSA and PSP. On the other hand, Marques and colleagues [42] as well as Hall and colleagues [32] revealed that patients with MSA/PSP/CBD can be accurately discerned from those with PD (AUC of 0.90 and 0.93, respectively). These findings may suggest that CSF NfL has potential in distinguishing PD from PD-plus syndromes rather than in differentiating among and between PD-plus syndromes.

Regarding blood NfL, three studies examined its diagnostic performance in MSA and PSP. Li and colleagues [39] reported an AUC of 0.802, whereas Schulz and colleagues [45] reported an AUC of 0.592. Of note, Marques and colleagues [42] reported an AUC of 0.91 in separating MSA/PSP from PD (following the trend reported in CSF studies).

Finally, the study of Schulz and colleagues compared patients with MSA directly to those with CBD and found a poor discriminatory potential for both CSF and circulating NfL (AUC < 0.600) [45].

### 3.3. Publication Bias

**Figure 8:**
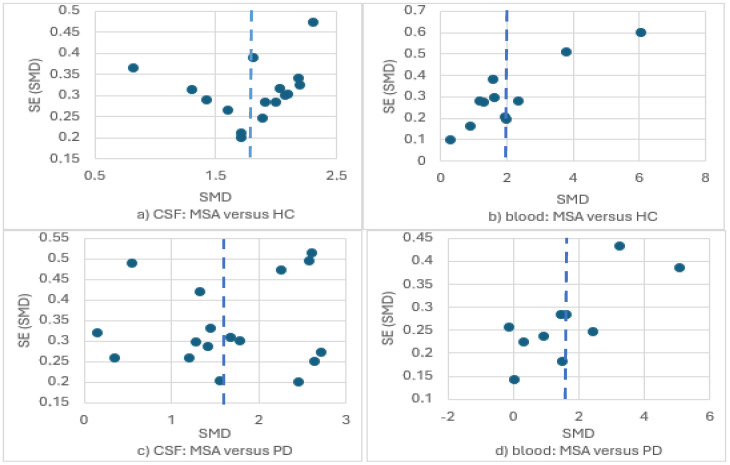
Funnel plots for the meta-analyses of NfL levels in (**a**) CSF: MSA versus HC; (**b**) blood: MSA versus HC; (**c**) CSF: MSA versus PD; (**d**) blood: MSA versus PD.

There was substantial publication bias in the context of the two out of the four meta-analyses with 10 or more studies (Figure 8). To be specific, the meta-analyses of circulating NfL in MSA versus HC and in MSA versus PD presented important publication bias with an over-publication of studies reporting greater though imprecise effect sizes (asymmetric inverted funnel). Only the funnel plot of the comparison of CSF NfL in MSA versus HC resembled the expected morphology of an inverted funnel. A less remarkable publication bias is probably present in the comparison of CSF NfL in MSA versus PD (no funnel morphology, asymmetric distribution of studies reporting greater though less precise effects). Overall, publication bias suggested that our estimations were probably larger than the true differences, particularly regarding circulating NfL.

## 4. Discussion

In recent years, efforts have been made to develop accurate biomarkers that may facilitate the clinical diagnosis of multiple system atrophy [56]. The objective of this study was to evaluate the role of CSF and circulating NfL. Regarding both CSF and circulating NfL, patients with MSA exhibit higher concentrations compared to HC, individuals with PD and LBD. An excellent diagnostic performance was reported for CSF and blood NfL in distinguishing MSA from HC, an outstanding performance in discriminating MSA from PD and a suboptimal performance in discerning MSA from LBD. However, fewer studies were published on the latter comparison. Regarding PSP and CBD, authors do not consistently report any differences in either CSF or circulating NfL in comparison with MSA. On the other hand, it appeared that NfL distinguished between PD and atypical parkinsonian syndromes (PSP, CBD, MSA) [57]. Of note, different authors report substantially variable CSF and blood NfL measurements as well as different optimum cut-points for discriminating between individuals with MSA and PD or HC.

NfL levels are a promising biomarker in the diagnosis of MSA. Its diagnostic potential is currently being explored by researchers in other neurodegenerative diseases, as well. NfL concentrations are consistently elevated in FTD patients and may serve as a diagnostic tool in the discrimination of FTD patients from HCs as well as from patients with LBD and primary psychiatric disorders (PPDs) [10]. Moreover, CSF and circulating NfL levels have been reported to distinguish between patients with ALS and HC, ALS mimics as well as other neurological disorders [58]. Also, NfL level has been proven extremely useful in multiple sclerosis as an aid for the diagnosis and particularly for the monitoring of disease progression [25,59]. Of interest, blood-based NfL level is also considered a non-specific marker of neural deterioration, and its levels have been associated with all-cause mortality in otherwise healthy adults [60].

Therefore, the question remains: why are MSA and potentially other Parkinson-plus syndromes related to greater levels of CSF and circulating NfL compared to PD? As the major structural component of neuraxons, higher NfL levels may reflect the more extensive and rapid degeneration characterizing these entities in relation to PD [61]. Although this pattern may extend to LBD, we report smaller differences in the sizes of the estimated effects. These undermined associations may be attributed to the binary nature of LBD [62]: in cases with long-standing PD and delayed conversion to PD dementia, NfL levels may correlate to those of idiopathic PD. In cases with DLB (which may be a rapidly progressive disorder, much alike Parkinson-plus syndromes), individuals may have greater NfL levels [63]. The clustering of these two entities (LBD) may be responsible for the intermediate sizes of the estimated differences. As a second potential explanation, higher NfL levels in MSA and other Parkinson-plus syndromes may reflect their more extensive subcortical (neuraxonal) degeneration in comparison with PD [64]. In this context, the occurrence of pyramidal symptoms has been also associated with greater NfL concentrations in extrapyramidal disorders [30].

Other emerging fluid biomarkers in the diagnosis of MSA include α-synuclein, tau, phosphorylated tau, β-amyloid-42 (Aβ-42) and GFAP [65]. Researchers have also proposed the use of fluid biomarker panels to increase the diagnostic accuracy of single biomarkers in the framework of MSA [39,66]. In addition to fluid biomarkers, imaging modalities (magnetic resonance imaging—MRI, single-photon emission computed tomography, positron emission tomography) are being studied to facilitate the diagnosis of MSA and its subclassification into MSA-P or MSA-C, depending on the patterns of cerebral atrophy, hypoperfusion and/or hypometabolism [4,67,68,69,70]. Overall, imaging and especially MRI studies as well as circulating biomarkers are gaining increasing interest in the study of neurodegeneration and will hopefully become established diagnostic tools that will replace the more invasive, more costly and less tolerable procedures, such as CSF and PET studies [71,72,73].

Retrieved studies exhibited substantial clinical heterogeneity, which was reflected in the analytical part of the article as well. Our qualitative approach emphasized the three main sources of heterogeneity: participant characteristics, disease duration and severity (Table 1). Retrieved studies included individuals with MSA over the 6th or 7th decade of life. The vast majority of the articles featured relatively matched HC and PD groups in terms of age. On the other hand, we observed significant heterogeneity in terms of disease duration with several authors recruiting individuals with MSA during the early disease course (first 2 years) and others during the later stages (later than 5 years). Of note, a number of studies involved individuals with MSA and PD with substantial between-group differences with respect to disease duration, up to 9 years. Moreover, substantial heterogeneity was observed in terms of disease severity. Of importance, apart from among-studies heterogeneity, several articles featured MSA and PD groups with significant between-group differences (even up to 20 degrees in the UPDRS part III scale). Finally, although the relevant effect cannot be quantified, heterogeneity is to be expected among different populations and especially among different laboratories. Overall, irrespective of the source of heterogeneity, different authors report substantially variable CSF and blood NfL measurements as well as different optimum cut-points for discriminating between individuals with MSA and PD or HC. These findings suggest that the standardization of processes (e.g., sampling and laboratory procedures, patient selection, i.e., diagnostic criteria, disease severity, duration, etc.) and the establishment of reference intervals and/or thresholds require further work.

We conducted a thorough analysis, involving a large sample of patients followed in tertiary and university hospital settings. In these settings, it more probable to establish an accurate diagnosis of MSA (or any other neurodegenerative disorder of the CNS; PD, LBD, PSP, CBD) and ensure accurate laboratory measurements (circulating and CSF NfL). However, our study exhibits several limitations as well. Despite the meticulous search of MEDLINE and SCOPUS, it is possible that we have missed a number of eligible publications. Moreover, data on LBD were gathered from a small number of studies, posing a question regarding the reliability of our findings. On the other hand, despite the small number of studies on PSP and CBD/S, the consistency of the reported associations is probably substantiating our findings. In addition to the above, data on the early and prediagnostic stages of MSA versus PD or LBD are rather deficient. As mentioned above, clinical heterogeneity is responsible for the quite imprecise estimations in the majority of our analyses. Of note, apart from the above-listed factors, there may be additional parameters contributing to the heterogeneity of the retrieved studies that were unaccounted for by the present analysis. Finally, retrieved studies presented important publication bias, especially in the context of circulating NfL; thus, our estimates are probably larger than the true between-group differences regarding circulating NfL. Overall, future research should prioritize the standardization of processes (so that different laboratories can obtain comparable NfL measurements), the more conscientious investigation of LBD (potentially the discrimination of DLB from PD dementia) and the implementation of these findings in earlier, less severe disease stages (even during the prediagnostic period).

## 5. Conclusions

In conclusion, this study revealed that CSF and circulating NfL concentrations are higher in MSA versus HC, PD and LBD but not versus PSP and CBD/S. Diagnostic metrics appear optimal in the distinction between MSA and PD. To put these findings into clinical context, the standardization of NfL measurements and the establishment of reference intervals and/or thresholds are essential.

## Figures and Tables

**Table 1:** Study characteristics.

Author—Publication Year	Settings	Participants per Group (Female%), Age in Years ± SD (Unless Stated Otherwise)	Diagnostic Criteria	Disease Duration in Years ± SD (Unless Stated Otherwise)	Disease Severity
Katzdobler 2024 [44] ^1,2,3,^*	MSA patients: recruited at the Department of Neurology, LMU University Hospital, LMU Munich and the Department of Neurology, Hannover Medical School. PD patients: recruited at the LMU Hospital and within the DESCRIBE and DANCER study of the German Center for Neurodegenerative Diseases (Germany)	Controls = 25 (44%), 62.4 ± 9.2 MSA = 47 (34%), 59.9 ± 7.8 PD = 24 (50%), 59.7 ± 8.7	MSA= second consensus statement on the diagnosis of multiple system atrophy PD = MDS-PD criteria	MSA = 4.0 ± 1.9 PD = 4.4 ± 3.3	MSA: UMSARS I + II = 43.8 ± 13.9 MSA: MDS-UPDRS III = 43.1 ± 17.8 PD: MDS-UPDRS III = 25.1 ± 12.2
Chen 2025 [36] ^1^	Patients from a cohort of Fujian Medical University Union Hospital-PD (China)	Controls = 30 (43%), 65.9 ± 6.0 MSA = 30 (46%), 64.5 ± 7.8 PD = 56 (35.7%), 65.0 ± 7.6 PSP = 33 (42.4%), 67.8 ± 5.9	MSA = second consensus statement on the diagnosis of multiple system atrophy PD = MDS-PD criteria PSP = MDS criteria All patients underwent brain magnetic resonance imaging or computed tomography scans	Months MSA = 40.3 ± 21.4 PD = 64.5 ± 34.9 PSP = 50.5 ± 29.8	
Liu 2024 [37] ^2^	Patients were enrolled from the CHina Initiative on Neurodegeneration in Aging (CHINA) cohort of patients with parkinsonism syndromes from the Movement Disorders Center of the Xuanwu Hospital at Capital Medical University in Beijing (China)	Controls = 54 (42.6%), 62.2 ± 6.9 MSA = 102 (40.2%), 60.9 ± 7.0 PD = 62 (48.4%), 65.3 ± 6.5	MSA = second consensus statement on the diagnosis of multiple system atrophy PD = MDS-PD criteria	Median (IQR) MSA = 2.5 (1.5–3.5) PD = 4.1 (1.9–7.4)	Median (IQR) MSA: UMSARS total = 33 (26–45.5)
Peng 2023 [38] ^1^	Patients were recruited from the Department of Neurology, Xiangya Hospital, Central South University and Changde First People’s Hospital, Hunan (China)	Controls = 211 (48%), 57.2 ± 9 MSA = 214 (44%), 58.2 ± 6.7 PD = 65 (44%), 62.9 ± 10.3	MSA = second consensus statement on the diagnosis of multiple system atrophy. All MSA patients were screened for exclusion of most common polyglutamine (polyQ) ataxia including spinocerebellar ataxia (SCA1, SCA2, SCA3, SCA6, SCA7, SCA17) and dentatorubral–pallidoluysian atrophy (DRPLA). PD = MDS-PD criteria	MSA = 2.4 ± 1.6 PD = 7.2 ± 4.3	MSA: UMSARS total = 38.14 ± 16.48
Guo 2023 [40] ^1^	Patients from Huashan Hospital, Fudan University and healthy controls from Qingdao Municipal Hospital (China)	Controls = 100 (56%), 59.50 ± 8.91 MSA = 73 (43%), 58.62 ± 7.85 PD = 28 (42%), 64.04 ± 9.33	MSA = second consensus statement on the diagnosis of multiple system atrophy PD = MDS-PD criteria	Months MSA = 19.48 ± 8.69 PD = 19.79 ± 10.92	
Tokutake 2022 [50] ^3^	Patients from Niigata University Hospital or at affiliated hospitals (Japan)	Controls = 20 (50%), 67 ± 5.9 MSA = 50 (50%), 65.9 ± 8.7	MSA = second consensus statement on the diagnosis of multiple system atrophy	MSA = 5.0 ± 3.0	UMSARS I = 20.8 ± 7.7 UMSARS II = 23.6 ± 9.2
Chelban 2022 [46] ^1,3^	Patients and controls from MSA specialist centers (UK (PROSPECT-M study), France, Spain, Germany and Russia)	Controls = 40 (50%), Median (IQR) = 64.5 (59–68) MSA-C = 106 (44%), Median (IQR) = 64 (56–59) MSA-P = 106 (56%), Median (IQR) = 64 (58–69)	MSA = second consensus statement on the diagnosis of multiple system atrophy A subset of participants also had volumetric T1-weighted 3 T MRI.	Median (IQR) MSA = 5 (3–6.5)	MSA-C: UMSARS total = 45.4 ± 16.5 MSA-P: UMSARS total = 46.6 ± 15.9
Li 2022 [39] ^1^	Patients from the movement disorders outpatient clinics of the General Hospital of Tianjin Medical University (China)	Controls = 33 (48%), 66.15 ± 4.75 MSA = 13 (43%), 63.11 ± 7.69 PD = 45 (11%), 66.15 ± 4.75 PSP = 8 (37.5%), 70.00 ± 7.31	MSA = second consensus statement on the diagnosis of multiple system atrophy PD = UK Parkinson’s Disease Society Brain Bank criteria PSP = National Institute for Neurological Disorders and Stroke/Society criteria for PSP	MSA = 2.22 ± 0.67 PD = 4.92 ± 2.47 PSP = 2.33 ± 1.86	MSA: Hoehn–Yahr stage = 3.63 ± 0.92 MSA: MDS-UPDRSIII = 42.54 ± 18.00 PD: Hoehn–Yahr stage = 1.31 ± 0.53 PD: MDS-UPDRSIII = 17.95 ± 10.70 PSP: Hoehn–Yahr stage = 3.17 ± 0.41 PSP: MDS-UPDRSIII = 25.75 ± 9.87
Quadalti 2021 [48] ^1,3^	Patients from Institute of Neurological Sciences of Bologna (ISNB) (Italy)	Controls = 72 (52.8%), 58.1 ± 10.1 MSA = 80 (53.8%), 61.1 ± 8.0 PD = 116 (30.2%), 59.9 ± 10.3 LBD = 64 (29.7%), 73.8 ± 5.7 PSP/CBS = 58 (46.5%), 71.2 ± 6.8	MSA = second consensus statement on the diagnosis of multiple system atrophy PD = MDS-PD criteria PSP/CBS = MDS criteria LBD = Fourth consensus report of the DLB Consortium Diagnostic investigations included, when available MRI, DaTSCAN, (MIBG)-SPECT, PSG	Months MSA = 51.2 ± 31.9 PD = 73.9 ± 52.6 LBD = 75.9 ± 90.9 PSP/CBS = 50.8 ± 31.2	MSA: Hoehn–Yahr = 3.1 ± 2.9 MSA: UPDRS III = 33.6 ± 11.9 PD: Hoehn–Yahr = 1.5 ± 0.6 PD: UPDRS III = 16.3 ± 8.5 LBD: Hoehn–Yahr = 2.1 ± 0.7 LBD: UPDRS III = 33.9 ± 11.2 PSP/CBS: Hoehn–Yahr = 2.7 ± 0.8 PSP/CBS: UPDRS III = 38.9 ± 25.4
Canaslan 2021 [52] ^3^	(Finland)	Controls = 35 (28%), 60.75 ± 13.39 MSA = 26 (53%), 65.15 ± 11.12 PD = 29 (31%), 66.38 ± 11.44 LBD = 23 (30%), 71.17 ± 10.13	MSA = first and second consensus statement on the diagnosis of multiple system atrophy PD = MDS-PD criteria LBD = fourth consensus report of the DLB Consortium		
Schulz 2021 [45] ^2,3^	Samples collected at the Paracelsus-Elena-Klinik, Kassel (Germany)	Controls = 20 (30%), 68.7 ± 6.38 MSA = 17 (23%), 66.47 ± 10.02 PD = 151 (34%), 69.36 ± 9.55 LBD = 45 (31%), 70.51 ± 6.51 CBD = 16 (50%), 69.25 ± 5.60 PSP = 38 (34%), 69.32 ± 6.57	MSA = second consensus statement on the diagnosis of multiple system atrophy PD = UK Parkinson’s Disease Society Brain Bank criteria LBD = fourth consensus report of the DLB Consortium PSP = MDS criteria CBD = Armstrong 2013 criteria for the diagnosis of corticobasal degeneration Also 1.5-tesla (MRI) to determine structural, quantitative levodopa testing, smell identification test		MSA: UPDRS III = 31 ± 6 PD: UPDRS III = 30 ± 17 PD: H&Y stage (median (IQR)) = 3 (1.5) LBD: UPDRS III = 27 ± 10
Singer 2020 [53] ^3^	Subjects were enrolled as a part of a prospective, longitudinal study of synucleinopathies (MSA, PD, and controls; Mayo Longitudinal Synucleinopathy Biomarker Study, NS092625) and prospective studies of dementia (DLB; Mayo Alzheimer’s Disease Research Center, AG016574 and Longitudinal Imaging Bio-markers of Disease Progression in DLB, NS100620) (USA)	Controls = 29 (52%), 58.5 ± 7.4 MSA = 62 (31%), 59.2 ± 6.8 PD = 16 (6%), 65.9 ± 6.0 LBD = 13 (0%), 66.8 ± 8.3	MSA = second consensus statement on the diagnosis of multiple system atrophy PD = MDS-PD criteria	Median (IQR) MSA = 3.2 (2.4–4.4) PD = 7.5 (5.4–10.8) LBD = 5.1 (4.2–8.5)	MSA: UMSARS total = 31.0 ± 9.1
Lin 2019 [51] ^1^	Patients were recruited from the National Taiwan University Hospital, a tertiary referral center in Taiwan.	Controls = 40 (45%), 68.6 ± 9.5 MSA = 22 (47.7%), 65.8 ± 7.4 PD = 116 (43.2%), 68.5 ± 11.2	MSA = second consensus statement on the diagnosis of multiple system atrophy PD = UK Parkinson’s Disease Society Brain Bank criteria	MSA = 5.2 ± 3.1 PD = 7.8 ± 6.5	MSA: Hoehn–Yahr stages (“off”) = 4.8 ± 2.4 MSA: UPDRS part III scores (“off”) = 32.6 ± 10.1 PD: Hoehn–Yahr stages (“off”) = 3.1 ± 1.2 PD: UPDRS part III scores (“off”) = 25.1 ± 10.6
Marques 2019 [42] ^2,3,^**	Patients from a study performed at the Radboud University Medical Center Nijmegen from the movement disorders outpatient clinic (the Netherlands)	Controls = 53 (45%), 57.5 ± 9.8 MSA = 22 (31.8%), 60.7 ± 7.1 PD = 55 (30.9%), 57 ± 10 PSP = 7 (42.8%), 68.9 ± 4.1	MSA = second consensus statement on the diagnosis of multiple system atrophy PD = UK Parkinson’s Disease Society Brain Bank criteria PSP = National Institute of Neurological Disorders and Stroke and Society for Progressive Supranuclear Palsy criteria for PSP Also (brain MRI, IBZM (iodobenzamide)-SPECT, anal sphincter EMG)	Months MSA = 33.9 ± 26.4 PD = 34.2 ± 26.3 PSP = 35.7 ± 19.2	MSA: H&Y score = 2.4 ± 1.0 MSA: UPDRS score = 29 ± 13.9 PD: H&Y score = 2.0 ± 0.6 PD: UPDRS score = 26.5 ± 11.9 PSP: H&Y score = 3.3 ± 0.7 PSP: UPDRS score = 35.9 ± 15.6
Hall 2018 [31] ^3^	Patients from the Clinic of Neurology, Skåne University Hospital, as part of the Swedish BioFINDER Study (Sweden)	Controls = 50 (56%), 65.3 ± 8.6 MSA = 24 (50%), 63.8 ± 8.0 PD = 131 (39%), 64.9 ± 10.6 PSP = 14 (64%), 71.5 ± 6.2	MSA = second consensus statement on the diagnosis of multiple system atrophy PD = NINDS criteria for PD PSP = National Institute of Neurological Disorders and Stroke and Society for Progressive Supranuclear Palsy criteria for PSP	MSA = 7.2 ± 4.5 PD = 5.5 ± 4.8 PSP = 5.7 ± 2.3	MSA: Hoehn–Yahr score = 4.1 ± 0.9 MSA: UPDRS part III scores = 45.1 ± 19.2 PD: Hoehn–Yahr score = 2.0 ± 0.8 PD: UPDRS part III scores = 17.1 ± 10.5 PSP: Hoehn–Yahr score = 4.1 ± 0.6 PSP: UPDRS part III scores = 45.4 ± 14.2
Hansson 2017 [35] ^3^	3 cohorts: Cohort 1 (Lund cohort) => In this convenience series, study participants were recruited at the Neurology Clinic, Skåne University Hospital, Lund (Sweden)	Controls = 53 (57%), 65 ± 8.4 MSA = 30 (43%), 64 ± 9.1 PD = 171 (37%), 65 ± 10.6 PSP = 19 (57%), 72 ± 5.9 CBD = 5 (80%), 69 ± 4.9	MSA = first consensus statement on the diagnosis of multiple system atrophy PD = National Institute of Neurological Disorders and Stroke Diagnostic Criteria for PD PSP = NINDS criteria for PSP CBD = Armstrong 2013 Criteria for the diagnosis of corticobasal degeneration	MSA = 6.5 ± 4.3 PD = 5.3 ± 5.2 PSP = 5.9 ± 2.2 CBD = 3.6 ± 1.3	MSA: Hoehn–Yahr score = 3.9 ± 1.1 MSA: UPDRS part III scores = 42 ± 21.1 PD: Hoehn–Yahr score = 1.9 ± 0.8 PD: UPDRS part III scores = 16.2 ± 10.3 PSP: Hoehn–Yahr score = 4.1 ± 0.7 PSP: UPDRS part III scores = 43 ± 15.1 CBD: Hoehn–Yahr score = 3.5 ± 1.7 CBD: UPDRS part III scores = 38.4 ± 26.7
	Cohort 2 = (London cohort) => enrolled at clinics at the National Hospital for Neurology and Neurosurgery, Queen Square (UK)	Controls = 26 (46%), 61 ± 9.5 MSA = 30 (47%), 65 ± 5.7 PD = 20 (45%), 65 ± 8.6 PSP = 29 (44%), 71 ± 6.1 CBD = 12 (83%), 71 ± 7.2	MSA = first consensus statement on the diagnosis of multiple system atrophy PD = UK Parkinson’s Disease Society Brain Bank criteria PSP = NINDS criteria for PSP CBD = Armstrong 2013 Criteria for the diagnosis of corticobasal degeneration	MSA = 4.3 ± 2.1 PD = 9.3 ± 6 PSP = 5.4 ± 2.8 CBD = 3.8 ± 2.2	MSA: Hoehn–Yahr score = 3.2 ± 1.0 PD: Hoehn–Yahr score = 2.5 ± 0.9 PSP: Hoehn–Yahr score = 3.9 ± 0.9 CBD: Hoehn–Yahr score = 3.2 ± 1
	Cohort 3 = (early disease cohort) => with early-stage disease (disease duration < 3 years) the Neurological Department, Sahlgrenska University Hospital, Göteborg (Sweden)	MSA = 28 (54%), 66 ± 9.4 PD = 53 (43%), 65 ± 12.2 CBD = 6 (100%), 65 ± 12.3 PSP = 22 (64%), 70 ± 8.2	MSA = first consensus statement on the diagnosis of multiple system atrophy PD = UK Parkinson’s Disease Society Brain Bank criteria CBD = Lang’s criteria for the diagnosis of corticobasal degeneration	MSA = 4.3 ± 2.1 PD= 9.3 ± 6 PSP = 5.4 ± 2.8 CBD = 3.8 ± 2.2	MSA: Hoehn–Yahr score = 3.1 ± 0.9 MSA: UPDRS part III scores = 27.6 ± 4 PD: Hoehn–Yahr score = 2 ± 0.6 PD: UPDRS part III scores = 23.4 ± 1.7 PSP: Hoehn–Yahr score = 3.2 ± 1 PSP: UPDRS part III scores = 29.6 ± 4 CBD: Hoehn–Yahr score = 2.8 ± 1
Herbert 2015 [43] ^3^	Patients referred to our tertiary movement disorder center at Radboud University Medical Centre, Nijmegen (The Netherlands)	Discovery Cohort Controls = 57 (35.1%), 57.0 ± 11.5 MSA = 27 (44.4%), 62.6 ± 9.0 PD = 36 (38.9), 60.1 ± 10.4	MSA = second consensus statement on the diagnosis of multiple system atrophy PD = the accuracy of diagnosis of parkinsonian syndromes in a specialist movement disorder service	Months, Median (Range) MSA = 43.7 (8–96) PD = 43.8 (6–158)	MSA: Hoehn–Yahr score = 2.7 ± 1.2 MSA: UPDRS = 32.5 ± 16.7 PD: Hoehn–Yahr score = 2.0 ± 0.60 PD: UPDRS = 30.3 ± 11.5
		Validation Cohort Controls = 56 (42.9%), 55.9 ± 11.1 MSA = 25 (32%), 62.5 ± 9.5 PD = 32 (28.1%) 56.5 ± 11.7	MSA = second consensus statement on the diagnosis of multiple system atrophy PD = the accuracy of diagnosis of parkinsonian syndromes in a specialist movement disorder service	Months, Median (Range) MSA = 38.0 (12–106) PD = 25.1 (6–84)	MSA: Hoehn–Yahr score = 2.6 ± 0.9 MSA: UPDRS = 32.4 ± 13.7 PD: Hoehn–Yahr score = 1.7 ± 0.4 PD: UPDRS = 20.4 ± 8.9
Magdalinou 2015 [47] ^3^	Patients from the movement disorders and cognitive clinics at the National Hospital for Neurology and Neurosurgery, Queen Square (UK)	Age = Mean (95%CI) Controls = 30 (50%), 59.8 (56.1–63.4) MSA = 31 (49.4%), 64.3 (62.2–66.4) PD = 31 (35.5%), 67.1 (64–70.2) PSP = 33 (42.4%), 70.3 (68.2–72.4) CBD = 14 (71.4%), 69.8 (65.5–74.1)	MSA = second consensus statement on the diagnosis of multiple system atrophy PSP = NINDS criteria for PSP All patients with parkinsonism underwent structural brain imaging (MRI/CT)	Median (IQR) MSA = 4 (3–6) PD = 8 (5–15) PSP = 5 (3–7) CBD = 3.5 (2–5)	Mean (95%CI) MSA: Hoehn–Yahr score = 3.2 (2.9–3.6) PD: Hoehn–Yahr score = 2.8 (2.4–3.1) PSP: Hoehn–Yahr score = 3.7 (3.4–4.1) CBD: Hoehn–Yahr score = 3.2 (2.8–3.6)
Bäckström 2015 [34] ^3^	Patients from a population-based incidence study of unselected cases of new onset idiopathic parkinsonism from a defined geographic catchment area (Sweden)	Age = Median Controls = 30 (46.7%), 69.6 MSA = 11 (27.3%), 72.9 PD = 99 (41.4%), 71.3 PSP = 12 (50%), 74.6	MSA = first consensus statement on the diagnosis of multiple system atrophy PD = UK Parkinson’s Disease Society Brain Bank criteria All patients with PD underwent N-ω-flouropropyl-2β-carbomethoxy-3β-(4-123I-i o dophenyl)nortropane, 123I-ioflupane (FP-CIT) single-photon emission computed tomography, and all demonstrated pathologic uptake. PSP = NINDS criteria for PSP	Months, Median MSA = 25 PD = 16 PSP = 13	Median MSA: modified Hoehn and Yahr Scale stage = 2.0 PD: modified Hoehn and Yahr Scale stage = 2.0 PSP: modified Hoehn and Yahr Scale stage = 2.5
Hall 2012 [32] ^3^	Patients from Skane University Hospital and Sahlgrenska University Hospital. (Sweden)	Age = Median (IQR) Controls = 107 (60.7%), 70 (63–76) MSA = 48 (54.2%) 64 (59–72) PD = 90 (34.4%) 63 (56–71) PSP = 45 (55.6%) 70 (64–74) LDB = 70 (31.4%) 74 (69–81) CBD = 12 (41.7%) 71 (66–76)	MSA = first consensus statement on the diagnosis of multiple system atrophy PD = National Institute of Neurological Disorders and Stroke diagnostic criteria for PD PSP = National Institute of Neurological Disorders and Stroke–Society for Progressive Supranuclear Palsy International Workshop CBD = NINDS criteria LBD = Fourth consensus report of the DLB Consortium		Median (IQR) MSA: Hoehn–Yahr score = 4 (3–5) PD: Hoehn–Yahr score = 2.5 (2–3) PSP: Hoehn–Yahr score = 4 (4–4) CBD: Hoehn–Yahr score = 4.5 (3–5)
Bech 2012 [49] ^3^	Patients from the outpatient Movement Disorders clinic at the Department of Neurology, Bispebjerg Hospital, and from the Memory Disorders Clinic, Department of Neurology, Rigshospitalet, Copenhagen University Hospitals. (Denmark)	Age = Median (range) MSA = 10 (40%), 60 (50–770) PD = 22 (40.9%), 56.5 (22–72) PSP = 10 (40%), 59.5 (55–65) LDB = 11 (18%), 70 (54–83) CBD = 3 (33%), 75 (59–76)	MSA = second consensus statement on the diagnosis of multiple system atrophy PD = UK Parkinson’s Disease Society Brain Bank clinical diagnostic criteria PSP = SIC Task Force Appraisal of Clinical Diagnostic Criteria LBD = third report of the DLB Consortium	Median (range) MSA = 5 (2–9) PD = 7 (2–22) PSP = 5 (3–10) LDB = 3 (2–8) CBD = 2 (1–7)	
Constantinescu 2010 [54] ^3^	Patients referred to the movement disorders team at the Neurology Department, Sahlgrenska University Hospital, Gothenburg (Sweden)	Controls = 59 (44%) MSA = 21 (62%) 57.4 PD = 10 (30%) 49.8 PSP = 14 (43%) 60.7 CBD = 5 (27%) 69.1	MSA = first consensus statement on the diagnosis of multiple system atrophy PD = UK Parkinson’s Disease Society Brain Bank criteria PSP = National Institute of Neurological Disorders and Stroke and Society for Progressive Supranuclear Palsy CBD = according to Lang et al.	MSA = 3.2 PD = 7.4 PSP = 2.9 CBD = 3.4	MSA: mean Hoehn–Yahr score = 3.3 PD: mean Hoehn–Yahr score = 2.9 PSP: mean Hoehn–Yahr score = 2.8 CBD: mean Hoehn–Yahr score = 3.3
Abdo 2007 [41] ^3^	Patients referred to the movement disorder clinic of the Department of Neurology at the Radboud University Nijmegen Medical Centre (The Netherlands)	Controls = 106, 52.8 ± 9.2 MSA-P = 19, 59.6 ± 6.6 PD = 31, 52.5 ± 10.8	MSA = SIC Task Force appraisal of clinical diagnostic criteria for parkinsonian disorders PD = UK Parkinson’s Disease Society Brain Bank criteria All patients had a brain MRI or CT-scan, some underwent an anal sphincter EMG and ancillary investigations to visualize the integrity of the pre-synaptic dopaminergic system ([123I]-CIT) or post-synaptic dopaminergic system (123IBZM-SPECT).	MSA = 4.1 ± 2.1 PD = 3.6 ± 2.8	Median (IQR) MSA: modified Hoehn–Yahr score = 2.5 (2.0–3.0) PD: modified Hoehn–Yahr score = 1.5 (1.5–2.0)
Holmberg 2001 [33] ^3^	Department of Neurology, University of Gothenburg, Sahlgrenska University Hospital (Sweden)	MSA = 36 (36%), 63.1 ± 9.4 PD = 35 (42%), 61.5 ± 10.5 PSP = 14 (35%), 68.5 ± 4.6	MSA = MDS criteria PD = UK Parkinson’s Disease Society Brain Bank criteria PSP = SIC Task Force Appraisal of Clinical Diagnostic Criteria MRI or a CT scan, a levodopa test with PLM registrations were performed	MSA = 4.7 ± 3.1 PD = 10.6 ± 6.9 PSP = 4.5 ± 2.5	
Holmberg 1998 [30] ^3^	Department of Neurology, University of Gothenburg, Sahlgrenska University Hospital (Sweden)	MSA = 10 (50%), 63.1 ± 9.2 PD = 19 (42%), 64.6 ± 7.8 PSP = 12 (66%), 66.5 ± 4.6	MSA = MDS criteria PD = UK Parkinson’s Disease Society Brain Bank criteria PSP = Criteria proposed by Golbe and Davis MRI or a CT scan	MSA = 5.4 ± 1.6 PD = 14.3 ± 9 PSP = 5.9 ± 3.7	

* For serum samples, corresponding plasma levels were calculated using a transformation equation based on matched serum and plasma samples; ** data from Herbert 2015—not included in the meta-analysis; ^1^ denotes plasma NfL measurements; ^2^ denotes serum NfL measurements; ^3^ denotes CSF NfL measurements; MSA: multiple system atrophy; PD: Parkinson’s disease; PSP: progressive supranuclear palsy; LBD: Lewy body dementia; CBD: corticobasal degeneration; SD: standard deviation; IQR: interquartile range; H&Y: Hoehn and Yahr scale; UPDRS: Unified Parkinson’s Disease Rating Scale; UMSARS: Unified Multiple System Atrophy Rating Scale; MDS: movement disorder society; UK: United Kingdom.

**Table 2:** Study results.

Author—Publication Year	Sample Type	MSA			CONTROLS			PD			Diagnostic Metrics (Cut-Off Values = pg/mL)
		*N*	Mean *	SD *	*N*	Mean *	SD *	*N*	Mean *	SD *	
Katzdobler 2024 [44]	Plasma	47	24.3	8.8	21	11.4	4.8	23	10.9	9.0	MSA vs. PD -AUC = 0.90 -Threshold = 14.07
	CSF	15	4580.1	2254.2	14	703.4	262.2	14	748.1	350.8	MSA vs. PD -AUC = 0.97 -Threshold = 1835.0
Chen 2025 [36]	Plasma	30	63.5	45.8	30	22.5	15.3	56	33.9	20.6	MSA vs. HC -AUC = 0.916 -Sensitivity = 93.3% -Specificity = 80.0% MSA vs. PD -AUC = 0.795 -Sensitivity = 83.3% -Specificity = 66.1%
Liu 2024 [37]	Serum	102	(Median) 356	(IQR) 260–455	54	(Median) 118	(IQR) 102–141	62	(Median) 165	(IQR) 121–217	MSA vs. HC -AUC = 0.935 -Threshold = 154.5 -Sensitivity = 99% -Specificity = 85.2% MSA vs. PD -AUC = 0.866 -Threshold = 223.5 -Sensitivity = 89.2% -Specificity = 80.6%
Peng 2023 [38]	Plasma	214	(Median) 35.94	(IQR) 6.99–171.6	211	(Median) 10.26	(IQR) 1.96–102.8	65	(Median) 15.95	(IQR) 3.5–172.51	MSA vs. HC -AUC = 0.946 -Threshold = 21.50-Sensitivity = 87.4% -Specificity = 92.4% MSA vs. PD -AUC = 0.849 -Threshold = 24.36-Sensitivity = 79.4% -Specificity = 78.5%
Guo 2023 [40]	Plasma	73	40.92	44.89	100	13.74	8.53	28	27.26	19.79	MSA vs. HC -AUC = 0.930 -Threshold = 22.68 -Sensitivity = 90% -Specificity = 86% MSA-P vs. PD -AUC = 0.662 -Threshold = 20.24 -Sensitivity = 94% -Specificity = 57%
Tokutake 2022 [50]	CSF	50	15,876	7779	20	2208	698.3				MSA vs. HC -AUC = 1 -Threshold = 3827 -Sensitivity = 100% -Specificity = 100%
Chelban 2022 [46]	Plasma	212	(Median) 39.9	(IQR) 27–48	40	(Median) 9.1	(IQR) 8.7–9.8				
	CSF	105	(Median) 4329	(IQR) 2577–5862	40	(Median) 560	(IQR) 420–855				
Li 2022 [39]	Plasma	13	86.53	33.74	33	16.00	5.18	45	20.43	14.09	MSA vs. HC -AUC = 1 -Sensitivity = 100% -Specificity = 100% MSA vs. PD -AUC = 0.983 -Sensitivity = 100% -Specificity = 95.6% MSA vs. PSP -AUC = 0.802 -Sensitivity = 78.3% -Specificity = 87.5%
Quadalti 2021 [48] ^1^	Plasma	54	(Median) 34.0	(IQR) 24.5–40.8	37	(Median) 8.9	(IQR) 6.3–12.2	62	(Median) 10.2	(IQR) 8.1–13.8	MSA vs. PD -AUC = 0.972 -Threshold = 17.2 -Sensitivity = 90.3% -Specificity = 96.4%
	CSF	68	(Median) 3098.0	(IQR) 2062.0–4314.0	35	(Median) 514.2	(IQR) 339.0–650.0	116	(Median) 566.5	(IQR) 424.0–694.0	MSA vs. PD -AUC = 0.991 -Threshold = 1196 -Sensitivity = 95.7% -Specificity = 100%
Canaslan 2021 [52] ^2^	CSF	26	3839	(S.E.M) 615.8	35	810	(S.E.M) 104.8	29	960.5	(S.E.M) 108.8	Combined MSA/LBD vs. PD -AUC = 0.87 Combined MSA/LBD vs. controls -AUC = 0.92
Schulz 2021 [45] ^3^	Serum	17	43.65	21.02	20	17.95	8.43	151	73.75	254.70	MSA vs. HC -AUC = 0.922 MSA vs. PD -AUC = 0.617 MSA vs. PSP -AUC = 0.592 MSA vs. LBD -AUC = 0.661 MSA vs. CBD -AUC = 0.535
	CSF	17	2886.59	1481.57	20	922.20	446.41	151	2130.91	2174.48	MSA vs. HC -AUC = 0.925 MSA vs. PD -AUC = 0.712 MSA vs. PSP -AUC = 0.577 MSA vs. LBD -AUC = 0.771 MSA vs. CBD -AUC = 0.574
Singer 2020 [53]	CSF										MSA vs. HC -AUC = 1 -Threshold = 1024–1225 -Sensitivity = 100% -Specificity = 100% MSA vs. PD/LBD -AUC = 0.97 -Threshold = 1400 -Sensitivity = 97% -Specificity = 90%
Lin 2019 [51]	Plasma	22	35.8	6.2	40	10.6	2.3	116	17.6	2.8	MSA vs. PD -AUC = 0.802 -Threshold = 24.06 -Sensitivity = 75.3% -Specificity = 80.4%
Marques 2019 [42] **	Serum	22	22.2	11	53	11.5	6.5	55	10.4	4.9	Combined MSA/PSP vs. HC -AUC = 0.88 -Sensitivity = 93% -Specificity = 72% Combined MSA/PSP vs. PD -AUC = 0.91 -Sensitivity = 86% -Specificity = 85%
	CSF	21	65,487	4138	32	1265	551	54	1249	666	Combined MSA/PSP vs. HC -AUC = 0.89 -Sensitivity = 75% -Specificity = 100% Combined MSA/PSP vs. PD -AUC = 0.90 -Sensitivity = 75% -Specificity = 98%
Hall 2018 [31]	CSF	24	3502.0	1932.7	50	887.2	724.0	131	870.2	649.1	
Hansson 2017 [35]	Lund cohort CSF	28	3435	1884	50	887	724	147	896	647	Blood NfL: PD vs. MSA -AUC = 0.91 -Sensitivity = 80% -Specificity = 91%
	London cohort CSF	29	3004	1438	26	638	291	5	2041	2908	Blood NfL: PD vs. MSA -AUC = 0.87 -Sensitivity = 97% -Specificity = 90%
Herbert 2015 [43]	Discovery Cohort CSF	27	4548	3206	57	1503	619	36	1350	915	
	Validation Cohort CSF	25	5938	4267	56	1290	664	32	1103	442	
Magdalinou 2015 [47]	CSF	31	(Median) 3024	(IQR) 1984–3818	30	(Median) 560	(IQR) 444–854	31	(Median) 966	(IQR) 637–1349	MSA vs. PSP -AUC = 0.66
Bäckström 2015 [34]	CSF	11	(Median) 1215	(IQR) 881–2052	30	(Median) 878	(IQR) 667–1120	99	(Median) 1143	(IQR) 706–1864	
Hall 2012 [32] ^4^	CSF	48	(Median) 4075	(IQR) 2270–7105	107	(Median) 860	(IQR) 610–1185	90	(Median) 980	(IQR) 670–1320	PD vs. PSP/MSA/CBD -AUC = 0.93
Bech 2012 [49] (data from Hu et al. [55])	CSF	10	1038	584				22	175	70	
Constantinescu 2010 [54]	CSF	21	(Median) 1207	(Range) 250–6030	59	(Median) 250	(Range) 250–710	10	(Median) 250	(Range) 250–347	
Abdo 2007 [41]	CSF	18	(Median) 33.4	(IQR) 18.3–62	36	(Median) 5.2	(IQR) 0–17.4	31	(Median) 6.7	(IQR) 5.4–7.7	MSA-P vs. PD -AUC = 0.92 -Threshold = 17.15 -Sensitivity = 83% -Specificity = 90%
Holmberg 2001 [33]	CSF	36	864	737				35	210	103	
Holmberg 1998 [30]	CSF	10	920	460				19	190	70	

* Unless stated otherwise; ** data from Herbert 2015—not included in the meta-analysis; *N*: number of participants; NfL values in LBD were provided by 4 studies: ^1^ plasma *N* = 33, median 17.1, IQR 14.7–34.2 and CSF *N* = 64, median 945, IQR 734.3–1253; ^2^ CSF *N* = 23, mean 2190, SEM 421.4; ^3^ serum *N* = 45, mean 34.88, SD 22.32 and CSF *N* = 45, mean 1615.29, SD 1139.03; ^4^ CSF *N* = 70, median, 1490 IQR 1180–2100; MSA: multiple system atrophy; PD: Parkinson’s disease; LBD: Lewy body dementia; PSP: progressive supranuclear palsy; CBD: corticobasal degeneration; SD: standard deviation; IQR: interquartile range.

## Data Availability

Not applicable.

## Supplementary Materials

The following supporting information can be downloaded at https://www.mdpi.com/article/10.3390/brainsci15030241/s1. The PRISMA 2020 Checklist. Reference [74] is cited in the supplementary materials.

## Abbreviations

The following abbreviations are used in this manuscript:

MSA	multiple system atrophy
MSA-P	multiple system atrophy parkinsonian type
MSA-C	multiple system atrophy cerebellar type
CSF	cerebrospinal fluid
NfL	neurofilament light chain
HC	healthy controls
PD	Parkinson’s disease
LBD	Lewy body dementia
DLB	dementia with Lewy bodies
PSP	progressive supranuclear palsy
CBD/S	corticobasal degeneration/syndrome
GCIs	glial cytoplasmic inclusions
SMD	standardized mean difference
CI	confidence interval
SD	standard deviation
IQR	interquartile range
RE	random effects
FE	fixed effects
AUC	area under the curve
p-tau	phosphorylated tau
GFAP	glial fibrillary acidic protein
MS	multiple sclerosis
AD	Alzheimer’s disease
FTD	frontotemporal dementia
ALS	amyotrophic lateral sclerosis
OPCA	olivopontocerebellar atrophy
QOC2	coenzyme Q2
REM	rapid eye movement
RBD	REM sleep behavior disorder
BD	bipolar disorder
MDD	major depressive disorder
PRISMA	Preferred Reporting Items for Systematic Reviews and Meta-Analyses
H&Y	Hoehn and Yahr scale
UPDRS	Unified Parkinson’s Disease Rating Scale
UMSARS	Unified Multiple System Atrophy Rating Scale
MDS	movement disorder society
MRI	magnetic resonance imaging
CT	computed tomography

## References

[1] Gilman S., Wenning G.K., Low P.A., Brooks D.J., Mathias C.J., Trojanowski J.Q., Wood N.W., Colosimo C., Dürr A., Fowler C.J. (2008). Second consensus statement on the diagnosis of multiple system atrophy. Neurology.

[2] Goh Y.Y., Saunders E., Pavey S., Rushton E., Quinn N., Houlden H., Chelban V. (2023). Multiple system atrophy. Pract. Neurol..

[3] Low P.A., Reich S.G., Jankovic J., Shults C.W., Stern M.B., Novak P., Tanner C.M., Gilman S., Marshall F.J., Wooten F. (2015). Natural history of multiple system atrophy in the USA: A prospective cohort study. Lancet Neurol..

[4] Fanciulli A., Wenning G.K. (2015). Multiple-System Atrophy. N. Engl. J. Med..

[5] Palma J.-A., Norcliffe-Kaufmann L., Kaufmann H. (2018). Diagnosis of multiple system atrophy. Auton. Neurosci. Basic Clin..

[6] Wan L., Zhu S., Chen Z., Qiu R., Tang B., Jiang H. (2023). Multidimensional biomarkers for multiple system atrophy: An update and future directions. Transl. Neurodegener..

[7] Gaetani L., Blennow K., Calabresi P., Di Filippo M., Parnetti L., Zetterberg H. (2019). Neurofilament light chain as a biomarker in neurological disorders. J. Neurol. Neurosurg. Psychiatry.

[8] Ramani S., Berard J.A., Walker L.A.S. (2021). The relationship between neurofilament light chain and cognition in neurological disorders: A scoping review. J. Neurol. Sci..

[9] van Zeggeren I.E., Ter Horst L., Heijst H., Teunissen C.E., van de Beek D., Brouwer M.C. (2022). Neurofilament light chain in central nervous system infections: A prospective study of diagnostic accuracy. Sci. Rep..

[10] Liampas I., Kyriakoulopoulou P., Karakoida V., Kavvoura P.A., Sgantzos M., Bogdanos D.P., Stamati P., Dardiotis E., Siokas V. (2024). Blood-Based Biomarkers in Frontotemporal Dementia: A Narrative Review. Int. J. Mol. Sci..

[11] Gallingani C., Carbone C., Tondelli M., Zamboni G. (2024). Neurofilaments Light Chain in Neurodegenerative Dementias: A Review of Imaging Correlates. Brain Sci..

[12] Quinn N. (2015). A short clinical history of multiple system atrophy. Clin. Auton. Res. Off. J. Clin. Auton. Res. Soc..

[13] Marmion D.J., Peelaerts W., Kordower J.H. (2021). A historical review of multiple system atrophy with a critical appraisal of cellular and animal models. J. Neural Transm..

[14] Papp M.I., Kahn J.E., Lantos P.L. (1989). Glial cytoplasmic inclusions in the CNS of patients with multiple system atrophy (striatonigral degeneration, olivopontocerebellar atrophy and Shy-Drager syndrome). J. Neurol. Sci..

[15] Ubhi K., Low P., Masliah E. (2011). Multiple System Atrophy: A Clinical and Neuropathological Perspective. Trends Neurosci..

[16] Valera E., Spencer B., Mott J., Trejo M., Adame A., Mante M., Rockenstein E., Troncoso J.C., Beach T.G., Masliah E. (2017). MicroRNA-101 Modulates Autophagy and Oligodendroglial Alpha-Synuclein Accumulation in Multiple System Atrophy. Front. Mol. Neurosci..

[17] Leńska-Mieciek M., Madetko-Alster N., Alster P., Królicki L., Fiszer U., Koziorowski D. (2023). Inflammation in multiple system atrophy. Front. Immunol..

[18] Campese N., Fanciulli A., Stefanova N., Haybaeck J., Kiechl S., Wenning G.K. (2021). Neuropathology of multiple system atrophy: Kurt Jellinger`s legacy. J. Neural Transm..

[19] McKay J.H., Cheshire W.P. (2018). First symptoms in multiple system atrophy. Clin. Auton. Res..

[20] Schrag A., Bohlken J., Kostev K. (2022). Pre-diagnostic presentations of Multiple System Atrophy case control study in a primary care dataset. Park. Relat. Disord..

[21] Tada M., Onodera O., Tada M., Ozawa T., Piao Y.-S., Kakita A., Takahashi H., Nishizawa M. (2007). Early development of autonomic dysfunction may predict poor prognosis in patients with multiple system atrophy. Arch. Neurol..

[22] Roncevic D., Palma J.-A., Martinez J., Goulding N., Norcliffe-Kaufmann L., Kaufmann H. (2014). Cerebellar and parkinsonian phenotypes in multiple system atrophy: Similarities, differences and survival. J. Neural Transm..

[23] Koga S., Aoki N., Uitti R.J., van Gerpen J.A., Cheshire W.P., Josephs K.A., Wszolek Z.K., Langston J.W., Dickson D.W. (2015). When DLB, PD, and PSP masquerade as MSA: An autopsy study of 134 patients. Neurology.

[24] Andersson E., Janelidze S., Lampinen B., Nilsson M., Leuzy A., Stomrud E., Blennow K., Zetterberg H., Hansson O. (2020). Blood and cerebrospinal fluid neurofilament light differentially detect neurodegeneration in early Alzheimer’s disease. Neurobiol. Aging.

[25] Freedman M.S., Gnanapavan S., Booth R.A., Calabresi P.A., Khalil M., Kuhle J., Lycke J., Olsson T. (2024). Consortium of Multiple Sclerosis Centers. Guidance for use of neurofilament light chain as a cerebrospinal fluid and blood biomarker in multiple sclerosis management. eBioMedicine.

[26] Bavato F., Barro C., Schnider L.K., Simrén J., Zetterberg H., Seifritz E., Quednow B.B. (2024). Introducing neurofilament light chain measure in psychiatry: Current evidence, opportunities, and pitfalls. Mol. Psychiatry.

[27] Hozo S.P., Djulbegovic B., Hozo I. (2005). Estimating the mean and variance from the median, range, and the size of a sample. BMC Med. Res. Methodol..

[28] Wan X., Wang W., Liu J., Tong T. (2014). Estimating the sample mean and standard deviation from the sample size, median, range and/or interquartile range. BMC Med. Res. Methodol..

[29] Teresa Greco G.B.-Z., Rin A.Z. (2015). How to impute study-specific standard deviations in meta-analyses of skewed continuous endpoints?. World J. Meta-Anal..

[30] Holmberg B., Rosengren L., Karlsson J., Johnels B. (1998). Increased cerebrospinal fluid levels of neurofilament protein in progressive supranuclear palsy and multiple-system atrophy compared with Parkinson’s disease. Mov. Disord..

[31] Hall S., Janelidze S., Surova Y., Widner H., Zetterberg H., Hansson O. (2018). Cerebrospinal fluid concentrations of inflammatory markers in Parkinson’s disease and atypical parkinsonian disorders. Sci. Rep..

[32] Hall S., Öhrfelt A., Constantinescu R., Andreasson U., Surova Y., Bostrom F., Nilsson C., Håkan W., Decraemer H., Någga K. (2012). Accuracy of a Panel of 5 Cerebrospinal Fluid Biomarkers in the Differential Diagnosis of Patients With Dementia and/or Parkinsonian Disorders. Arch. Neurol..

[33] Holmberg B., Johnels B., Ingvarsson P., Eriksson B., Rosengren L. (2001). CSF-neurofilament and levodopa tests combined with discriminant analysis may contribute to the differential diagnosis of Parkinsonian syndromes. Park. Relat. Disord..

[34] Bäckström D.C., Eriksson Domellöf M., Linder J., Olsson B., Öhrfelt A., Trupp M., Zetterberg H., Blennow K., Forsgren L. (2015). Cerebrospinal Fluid Patterns and the Risk of Future Dementia in Early, Incident Parkinson Disease. JAMA Neurol..

[35] Hansson O., Janelidze S., Hall S., Magdalinou N., Lees A.J., Andreasson U., Norgren N., Linder J., Forsgren L., Constantinescu R. (2017). Blood-based NfL: A biomarker for differential diagnosis of parkinsonian disorder. Neurology.

[36] Chen Y., Huang J., Li Y., Chen X., Ye Q. (2025). Diagnostic value of six plasma biomarkers in progressive supranuclear palsy, multiple system atrophy, and Parkinson’s disease. Clin. Chim. Acta.

[37] Liu M., Cai Y., Pan J., Wang T., Li Y., Yu Q., Mao W., Chan P. (2024). Serum neurofilament light chain as a diagnostic and prognostic biomarker in multiple system atrophy: A prospective cohort study. J. Neurol..

[38] Peng L., Wan L., Liu M., Long Z., Chen D., Yuan X., Tang Z., Fu Y., Zhu S., Lei L. (2023). Diagnostic and prognostic performance of plasma neurofilament light chain in multiple system atrophy: A cross-sectional and longitudinal study. J. Neurol..

[39] Li Q., Li Z., Han X., Shen X., Wang F., Bai L., Li Z., Zhang R., Wang Y., Zhu X. (2022). A Panel of Plasma Biomarkers for Differential Diagnosis of Parkinsonian Syndromes. Front. Neurosci..

[40] Guo Y., Shen X.-N., Huang S.-Y., Chen S.-F., Wang H.-F., Zhang W., Zhang Y.R., Cheng W., Cui M., Dong Q. (2023). Head-to-head comparison of 6 plasma biomarkers in early multiple system atrophy. NPJ Park. Dis..

[41] Abdo W.F., Bloem B.R., Van Geel W.J., Esselink R.A.J., Verbeek M.M. (2007). CSF neurofilament light chain and tau differentiate multiple system atrophy from Parkinson’s disease. Neurobiol. Aging.

[42] Marques T.M., van Rumund A., Oeckl P., Kuiperij H.B., Esselink R.A.J., Bloem B.R., Otto M., Verbeek M.M. (2019). Serum NFL Discriminates Parkinson Disease from Atypical Parkinsonisms. Neurology.

[43] Herbert M.K., Aerts M.B., Beenes M., Norgren N., Esselink R.A.J., Bloem B.R., Kuiperij H.B., Verbeek M.M. (2015). CSF Neurofilament Light Chain but not FLT3 Ligand Discriminates Parkinsonian Disorders. Front. Neurol..

[44] Katzdobler S., Nübling G., Klietz M., Fietzek U.M., Palleis C., Bernhardt A.M., Wegner F., Huber M., Rogozinski S., Schneider L.S. (2024). GFAP and NfL as fluid biomarkers for clinical disease severity and disease progression in multiple system atrophy (MSA). J. Neurol..

[45] Schulz I., Kruse N., Gera R.G., Kremer T., Cedarbaum J., Barbour R., Zago W., Schade S., Otte B., Bartl M. (2021). Systematic Assessment of 10 Biomarker Candidates Focusing on α-Synuclein-Related Disorders. Mov. Disord. Off. J. Mov. Disord. Soc..

[46] Chelban V., Nikram E., Perez-Soriano A., Wilke C., Foubert-Samier A., Vijiaratnam N., Guo T., Jabbari E., Olufodun S., Gonzalez M. (2022). Neurofilament light levels predict clinical progression and death in multiple system atrophy. Brain.

[47] Magdalinou N.K., Paterson R.W., Schott J.M., Fox N.C., Mummery C., Blennow K., Bhatia K., Morris H.R., Giunti P., Warner T.T. (2015). A panel of nine cerebrospinal fluid biomarkers may identify patients with atypical parkinsonian syndromes. J. Neurol. Neurosurg. Psychiatry.

[48] Quadalti C., Calandra-Buonaura G., Baiardi S., Mastrangelo A., Rossi M., Zenesini C., Giannini G., Candelise N., Sambati L., Polischi B. (2021). Neurofilament light chain and α-synuclein RT-QuIC as differential diagnostic biomarkers in parkinsonisms and related syndromes. NPJ Park. Dis..

[49] Bech S., Hjermind L.E., Salvesen L., Nielsen J.E., Heegaard N.H.H., Jørgensen H.L., Rosengren L., Blennow K., Zetterberg H., Winge K. (2012). Amyloid-related biomarkers and axonal damage proteins in parkinsonian syndromes. Park. Relat. Disord..

[50] Tokutake T., Kasuga K., Tsukie T., Ishiguro T., Shimohata T., Onodera O., Ikeuchi T. (2022). Clinical correlations of cerebrospinal fluid biomarkers including neuron-glia 2 and neurofilament light chain in patients with multiple system atrophy. Park. Relat. Disord..

[51] Lin C.-H., Li C.-H., Yang K.-C., Lin F.-J., Wu C.-C., Chieh J.-J., Chiu M.J. (2019). Blood NfL: A biomarker for disease severity and progression in Parkinson disease. Neurology.

[52] Canaslan S., Schmitz M., Villar-Piqué A., Maass F., Gmitterová K., Varges D., Lingor P., Llorens F., Hermann P., Zerr I. (2021). Detection of cerebrospinal fluid neurofilament light chain as a marker for alpha-synucleinopathies. Front. Aging Neurosci..

[53] Singer W., Schmeichel A.M., Shahnawaz M., Schmelzer J.D., Boeve B.F., Sletten D.M., Gehrking T.L., Gehrking J.A., Olson A.D., Savica R. (2020). Alpha-Synuclein Oligomers and Neurofilament Light Chain in Spinal Fluid Differentiate Multiple System Atrophy from Lewy Body Synucleinopathies. Ann. Neurol..

[54] Constantinescu R., Rosengren L., Johnels B., Zetterberg H., Holmberg B. (2010). Consecutive analyses of cerebrospinal fluid axonal and glial markers in Parkinson’s disease and atypical parkinsonian disorders. Park. Relat. Disord..

[55] Hu X., Yang Y., Gong D. (2017). Cerebrospinal fluid levels of neurofilament light chain in multiple system atrophy relative to Parkinson’s disease: A meta-analysis. Neurol. Sci. Off. J. Ital. Neurol. Soc. Ital. Soc. Clin. Neurophysiol..

[56] Krismer F., Fanciulli A., Meissner W.G., Coon E.A., Wenning G.K. (2024). Multiple system atrophy: Advances in pathophysiology, diagnosis, and treatment. Lancet Neurol..

[57] Angelopoulou E., Bougea A., Papadopoulos A., Georgakis M.K., Stefanis L. (2021). CSF and circulating NfL as biomarkers for the discrimination of Parkinson disease from atypical parkinsonian syndromes: Meta-analysis. Neurol. Clin. Pract..

[58] Sferruzza G., Bosco L., Falzone Y.M., Filippi M., Riva N. (2022). Neurofilament light chain as a biological marker for amyotrophic lateral sclerosis: A meta-analysis study. Amyotroph. Lateral Scler. Front. Degener..

[59] Kouchaki E., Dashti F., Mirazimi S.M.A., Alirezaei Z., Jafari S.H., Hamblin M.R., Mirzaei H. (2021). Neurofilament light chain as a biomarker for diagnosis of multiple sclerosis. EXCLI J..

[60] Nguyen A.D., Malmstrom T.K., Aggarwal G., Miller D.K., Vellas B., Morley J.E. (2022). Serum neurofilament light levels are predictive of all-cause mortality in late middle-aged individuals. eBioMedicine.

[61] Wang S.Y., Chen W., Xu W., Li J.Q., Hou X.H., Ou Y.N., Yu J.T., Tan L. (2019). Neurofilament light chain in cerebrospinal fluid and blood as a biomarker for neurodegenerative diseases: A systematic review and meta-analysis. J. Alzheimers Dis..

[62] Liampas I., Kyriakoulopoulou P., Siokas V., Tsiamaki E., Stamati P., Kefalopoulou Z., Chroni E., Dardiotis E. (2024). Apolipoprotein E gene in α-synucleinopathies: A narrative review. Int. J. Mol. Sci..

[63] Gaig C., Valldeoriola F., Gelpi E., Rey M.J., Martí M.J., Graus F., Tolosa E. (2011). Rapidly progressive diffuse Lewy body disease. Mov. Disord..

[64] Tsukamoto K., Matsusue E., Kanasaki Y., Kakite S., Fujii S., Kaminou T., Ogawa T. (2012). Significance of apparent diffusion coefficient measurement for the differential diagnosis of multiple system atrophy, progressive supranuclear palsy, and Parkinson’s disease: Evaluation by 3.0-T MR imaging. Neuroradiology.

[65] Laurens B., Constantinescu R., Freeman R., Gerhard A., Jellinger K., Jeromin A., Krismer F., Mollenhauer B., Schlossmacher M.G., Shaw L. (2015). Fluid biomarkers in multiple system atrophy: A review of the MSA Biomarker Initiative. Neurobiol. Dis..

[66] Taha H.B., Hornung S., Dutta S., Fenwick L., Lahgui O., Howe K., Elabed N., Del Rosario I., Wong D.Y., Duarte Folle A. (2023). Toward a biomarker panel measured in CNS-originating extracellular vesicles for improved differential diagnosis of Parkinson’s disease and multiple system atrophy. Transl. Neurodegener..

[67] Dash S.K., Kamble N., Stezin A., Yadav R., Netravathi M., Saini J., Pal P. (2024). Imaging Markers of Multiple System Atrophy and Their Association With Disease Severity: A Cross-Sectional Study. Cureus.

[68] Kim H.-J., Jeon B., Fung V.S.C. (2016). Role of Magnetic Resonance Imaging in the Diagnosis of Multiple System Atrophy. Mov. Disord. Clin. Pract..

[69] Cilia R., Marotta G., Benti R., Pezzoli G., Antonini A. (2005). Brain SPECT imaging in multiple system atrophy. J. Neural Transm..

[70] Zhao P., Zhang B., Gao S., Li X. (2020). Clinical features, MRI, and 18F-FDG-PET in differential diagnosis of Parkinson disease from multiple system atrophy. Brain Behav..

[71] Liampas I., Siokas V., Zoupa E., Kyriakoulopoulou P., Stamati P., Provatas A., Tsouris Z., Tsimourtou V., Lyketsos C.G., Dardiotis E. (2024). Neuropsychiatric symptoms and white matter hyperintensities in older adults without dementia. Int. Psychogeriatr..

[72] Liampas I., Siokas V., Stamati P., Kyriakoulopoulou P., Tsouris Z., Zoupa E., Folia V., Lyketsos C.G., Dardiotis E. (2024). Neuropsychiatric Symptoms Associated With Frontotemporal Atrophy in Older Adults Without Dementia. Int. J. Geriatr. Psychiatry.

[73] Antonioni A., Raho E.M., Manzoli L., Koch G., Flacco M.E., Di Lorenzo F. (2025). Blood phosphorylated Tau181 reliably differentiates amyloid-positive from amyloid-negative subjects in the Alzheimer’s disease continuum: A systematic review and meta-analysis. Alzheimers Dement..

[74] Page M.J., McKenzie J.E., Bossuyt P.M., Boutron I., Hoffmann T.C., Mulrow C.D., Shamseer L., Tetzlaff J.M., Akl E.A., Brennan S.E. (2021). The PRISMA 2020 statement: An updated guideline for reporting systematic reviews. BMJ.

